# Comparative study of post-growth annealing of Cu(hfac)_2_, Co_2_(CO)_8_ and Me_2_Au(acac) metal precursors deposited by FEBID

**DOI:** 10.3762/bjnano.9.11

**Published:** 2018-01-09

**Authors:** Marcos Vinicius Puydinger dos Santos, Aleksandra Szkudlarek, Artur Rydosz, Carlos Guerra-Nuñez, Fanny Béron, Kleber Roberto Pirota, Stanislav Moshkalev, José Alexandre Diniz, Ivo Utke

**Affiliations:** 1 Institute of Physics Gleb Wataghin, University of Campinas, Rua Sérgio Buarque de Holanda 777 Cidade Universitária, 13083-859, Campinas-SP, Brazil; 2 Faculty of Electrical and Computing Engineering and Center for Semiconductor Components and Nanotechnologies, University of Campinas, Rua Pandiá Calógeras 90, Cidade Universitária, 13083-870, Campinas-SP, Brazil; 3 Laboratory for Mechanics of Materials and Nanostructures, Swiss Federal Laboratories for Materials Science and Technology (EMPA), Feuerwerkerstrasse 39, 3602 Thun, Switzerland; 4 AGH University of Science and Technology, Academic Centre for Materials and Nanotechnology, al. A. Mickiewicza 30, 30-059 Krakow, Poland; 5 AGH University of Science and Technology, Faculty of Computer Science, Electronics and Telecommunications, Av. Mickiewicza 30, 30-059 Krakow, Poland

**Keywords:** copper, gold, cobalt, focused-electron-beam-induced deposition, noble metal, non-noble metals, post-growth annealing

## Abstract

Non-noble metals, such as Cu and Co, as well as noble metals, such as Au, can be used in a number modern technological applications, which include advanced scanning-probe systems, magnetic memory and storage, ferroelectric tunnel junction memristors, metal interconnects for high performance integrated circuits in microelectronics and nano-optics applications, especially in the areas of plasmonics and metamaterials. Focused-electron-beam-induced deposition (FEBID) is a maskless direct-write tool capable of defining 3-dimensional metal deposits at nanometre scale for above applications. However, codeposition of organic ligands when using organometallic precursors is a typical problem that limits FEBID of pure metal nanostructures. In this work, we present a comparative study using a post-growth annealing protocol at 100, 200, and 300 °C under high vacuum on deposits obtained from Co_2_(CO)_8_, Cu(II)(hfac)_2_, and Me_2_Au(acac) to study improvements on composition and electrical conductivity. Although the as-deposited material was similar for all precursors, metal grains embedded in a carbonaceous matrix, the post-growth annealing results differed. Cu-containing deposits showed the formation of pure Cu nanocrystals at the outer surface of the initial deposit for temperatures above 100 °C, due to the migration of Cu atoms from the carbonaceous matrix containing carbon, oxygen, and fluorine atoms. The average size of the Cu crystals doubles between 100 and 300 °C of annealing temperature, while the composition remains constant. In contrast, for Co-containing deposits oxygen release was observed upon annealing, while the carbon content remained approximately constant; the cobalt atoms coalesced to form a metallic film. The as-deposited Au-containing material shows subnanometric grains that coalesce at 100 °C, maintaining the same average size at annealing temperatures up to 300 °C. Raman analysis suggests that the amorphous carbonaceous matrix of the as-written Co, Cu and Au deposits turned into nanocrystalline graphite with comparable crystal sizes of 12–14 nm at 300 °C annealing temperature. However, we observed a more effective formation of graphite clusters in Co- than in Cu- and Au-containing deposits. The graphitisation has a minor influence on the electrical conductivity improvements of Co–C deposits, which is attributed to the high as-deposited Co content and the related metal grain percolation. On the contrary, electrical conductivity improvements by factors of 30 and 12 for, respectively, Cu–C and Au–C deposits with low metal content are mainly attributed to the graphitisation. This relatively simple vacuum-based post-growth annealing protocol may be useful for other precursors as it proved to be efficient in reliably tuning the electrical properties of as-deposited FEBID materials. Finally, a H_2_-assisted gold purification protocol is demonstrated at temperatures around 300 °C by fully removing the carbon matrix and drastically reducing the electrical resistance of the deposit.

## Introduction

Focused-electron-beam-induced deposition (FEBID) constitutes a well-established maskless nanopattering technique. It is based on the local dissociation of adsorbates upon the irradiation with electrons, combining the advantages of a direct-write process with the depositing possibility of a number of geometries at nanometric scale [1–11]. The deposition mechanism consists of the delivery of gas molecules into a scanning electron microscope (SEM) chamber by a gas injection system (GIS), and the subsequent reversible physiosorption of these molecules on the substrate surface. Part of the energy, from both the primary electron beam and the secondary electrons generated in the vicinity of the impinging primary beam, is transferred to the adsorbates. The dissociation yields both volatile ligand fragments (pumped away) and deposited non-volatile products (such as metals) [8–9,12–13].

FEBID has been recently used to define nanodevices for several applications, such as gas [14–15], strain [16], magnetic [12,17] and thermal sensors [18], besides nano-antennas as probes for scanning near-field optical microscopy (SNOM) [19]. Other applications comprising superconducting [20] and plasmonic [21] structures, nanoalloys for nanoelectronic applications [22], ferromagnetic nanostructures for magnetic logic and memory [23–25], as well as functional core–shell ferromagnetic nanowires with improved magnetic properties [26] have already been realised through this technique. It is worth mentioning that the superior conductivity of Cu, compared to other non-noble metals, makes its localised direct-write deposition attractive for applications in high-performance integrated circuits and nanoelectronics [13,27]. Similarly, Au nanostructures are promising materials in nanoplasmonics, biomedical applications, electrochemical sensing, as well as contacts for carbon-based devices [28]. Furthermore, the possibility of depositing 3D nanostructures with high aspect ratio makes FEBID an adequate tool for the fabrication of advanced scanning-probe systems, as well as high-resolution ferromagnetic probes for magnetic force microscopy (MFM) [29–31].

Nevertheless, the incomplete dissociation of organometallic precursor adsorbate molecules usually leads to codeposition of organic compounds into the metal deposits [3,8,12,32–35]. This unwanted deposition degrades the electrical transport properties of the deposit, thus limiting the applicability of the FEBID material.

Several purification methods have been successfully employed to remove the residual carbon content from Pt and Au deposits. They consist of post-growth electron-beam-irradiation under a local O_2_ or H_2_O ambient [3,32–34,36–39], in situ direct growth under O_2_ flow [35,40], as well as laser-assisted thermal dissociation [41–43]. In the case of Pt–C, the catalytic properties of the Pt nanoparticles facilitate the dissociation process of molecular oxygen, thus increasing the efficiency in removing the carbonaceous matrix [35]. With respect to Au deposits, a content of 91 atom % was achieved by using H_2_O as an oxidative carbon remover during direct-write deposition of Me_2_Au(tfac) [38]. Moreover, focused-electron-beam-induced curing (FEBIC), combined with O_2_ plasma-assistance, has been used for enhancing the Au content from Me_2_Au(tfac) [28].

However, such oxidising methods are not utilized in depositions of non-noble metals in order to avoid oxidation of the metals. For W–C deposits, an electrical conductivity improvement of one order of magnitude was obtained using a genetic algorithm to optimize the deposition parameters [44]. In addition, high-purity W deposits (up to 95 atom % relative to carbon) were obtained by means of impact-enhanced desorption of residual organic ligands using a supersonic argon carrier gas jet to deliver the organometallic precursor [45]. Other works suggest the autocatalytic deposition of carbonyls, such as Fe(CO)_5_ [46–47] and Co_2_(CO)_8_ [48–49], to produce high-purity metallic deposits, while electron-beam irradiation can be used during a sequential exposure of the FEBID material to oxygen (favouring carbon release) and hydrogen (favouring metal oxide reduction) [50].

Furthermore, a Co content exceeding 75 atom % was recently achieved with the use of nitrogen as carrier gas, using a strategy to overcome the usual problem caused by temperature gradients along the GIS [51]. Additionally, direct-write processes with optimized parameters allowed for the deposition of high-purity Co deposits in combination with high lateral resolution [52–53]. Novel strategies have also been recently implemented to produce high-purity Cu nanodeposits using an aqueous solution precursor [54].

Moreover, the conventional post-growth annealing of FEBID deposits under vacuum has been reported as a promising protocol for tuning the non-noble metal content of a number of precursors [3,5,7–8,27]. The thermal energy delivered to the sample can cause desorption of volatile fragments, thus increasing the metal concentration [13]. In this work we compare the post-growth annealing in high vacuum of non-noble (Co–C, Cu–C) and noble (Au–C) FEBID materials. We present the potential of this protocol with respect to the aforementioned works for the fabrication of pure copper and gold nanocrystals embedded in a graphitic matrix, as well as cobalt–carbon nanocomposite films, from the as-prepared amorphous metal–C deposit. Finally, a new H_2_-assisted Au–C FEBID material purification at temperatures below 300 °C is presented, which results in pure Au crystals by fully removing the carbon matrix. This allows for electrical conductivity improvements by three orders of magnitude.

## Experimental

FEBID was used to separately obtain 35 nm thick Co*_x_*C*_y_*O*_z_*, 50 nm thick Cu*_x_*F*_y_*C*_z_*O*_z_* and 50 nm thick Au_x_C_y_O_z_ depositions. Each defined a rectangular pattern of 20 × 5 μm^2^ onto predefined 100 nm thick Au electrodes. These electrodes were obtained by standard UV lithography on phosphorus-doped Si wafers (8 mΩ·cm) with a 200 nm thick SiO_2_ layer for electrodes isolation. The substrate was kept at room temperature during all depositions, which were performed using a Hitachi S-3600 SEM with a tungsten filament, beam energy of 15 keV, 1.5 nA beam current, electron flux of 2.6 × 10^18^ s^−1^·cm^−2^, 10 μs dwell time per pixel, 9.6 ms refreshment time, serpentine scan and 10 nm pixel-to-pixel distance.

Dicobalt octacarbonyl [Co_2_(CO)_8_], bis(hexafluoroacetylacetonato)copper(II) [Cu(hfac)_2_, Cu(HC_5_O_2_F_6_)_2_] and dimethyl(acetylacetonato)gold(III) [Me_2_Au(acac), (CH_3_)_2_Au(C_5_H_7_O_2_)] were employed as precursors, with average fluxes of 4.1 × 10^18^, 2.9 × 10^17^ and 3.6 × 10^17^ molecules·s^−1^·cm^−2^, respectively. The precursors have been separately filled into their reservoirs inside a glove box in argon atmosphere, before being introduced on the substrate surface by heating up to 50 °C the GIS. Each precursor was used separately on different samples and experiments in order to avoid cross contamination. Values of about 1, 10, and 10 electrons per impinging precursor molecule, respectively, for Co_2_(CO)_8_, Cu(hfac)_2_ and Me_2_Au(acac), were inferred from the calculated electronic flux, which we supposed to be close to the electron-limited regime. The chamber pressure during FEBID experiments was maintained at around (4–6) × 10^−5^ mbar for all the three precursors. The vertical distance between the bottom portion of the GIS and the substrate surface was fixed at 200 μm, while the in-plane distance between the GIS and the deposit area was about 50 μm.

Post-growth annealing processes on FEBID deposits were performed in order to increase the metallic content. Annealing temperatures of 100, 200 and 300 °C were kept for 10 min, after a ramp rate for temperature stabilization of ca. 20 K·min^−1^. This process was carried out inside the SEM chamber using a dedicated custom-built heating stage, but in the absence of precursor flux, under a pressure of around 3 × 10^−5^ mbar. Furthermore, for the H_2_-assisted purification of Au–C deposits, a 200 ppm H_2_ atmosphere was utilized while the sample was heated from room temperature to 360 °C over a period of 6.5 h. The electrical resistance was monitored in situ using a two-wire setup.

## Results and Discussion

### Deposit morphology characterisation

An atomic force microscope (AFM, NT-MDT NTEGRA spectra) was used to determine the thickness and the volume of the FEBID deposits as a function of the annealing temperature. We observed that the deposition rates per rectangle area of 100 μm^2^ were similar for Cu–C and Au–C (ca. 1.7 nm·min^−1^), while they were slightly lower for Co–C (ca. 1.4 nm·min^−1^), being comparable to typical atomic layer deposition (ALD) processes.

Measurements performed on deposits after annealing at 300 °C indicate shrinkage with respect to the as-deposited thickness. While this shrinkage is low for Cu–C and Au–C deposits (below 5%), Co–C deposits exhibit again a different value, with an observed shrinkage of 7–10%. We attribute the thickness fluctuations to process parameters, such as variations in the electron beam flux due to astigmatism and focus refinements, as well as variations in the molecular flux due to thermal gradients in the GIS.

### Optical microscopy and SEM characterisations

**Figure 1:**
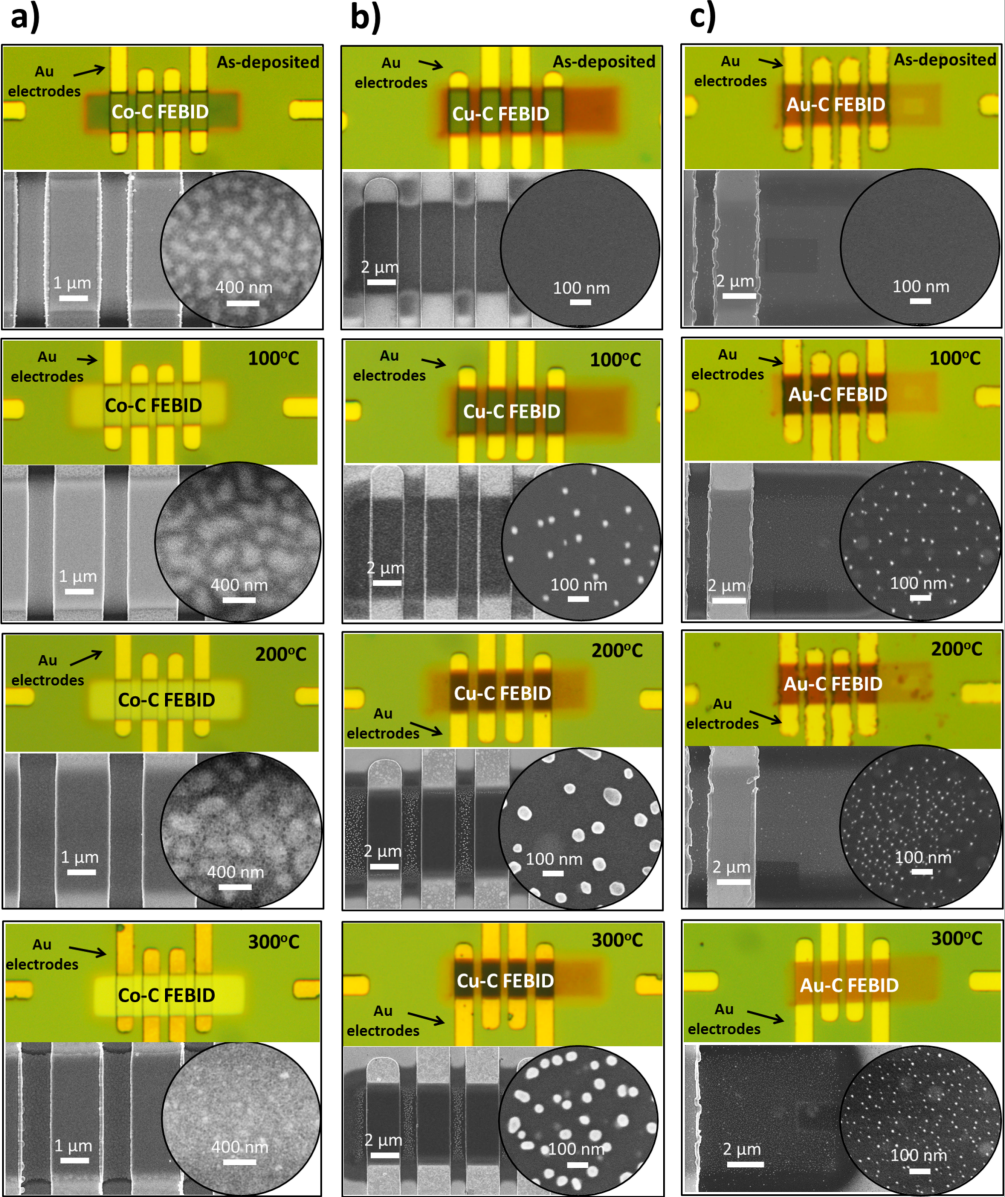
Optical microscopy images showing the 200 nm SiO_2_/Si substrate and gold electrodes together with (a) 35 nm thick Co–C, (b) 50 nm thick Cu–C and (c) 50 nm thick Au–C FEB-induced deposits of 20 × 5 μm^2^ area. The post-growth annealing temperatures are indicated. The corresponding SEM images (bottom) show the deposits at higher magnification. Note that the Co–C deposit exhibits changing colour and increasing reflectivity at visible wavelengths with annealing temperature, while only slight changes are observed in Cu–C and Au–C deposits.

In a first step, we observed the modifications induced by the annealing process in the deposited material through optical characterization. It was performed in a conventional Leica optical microscope with a maximum magnification of 1000× in the absence of filters. We attribute the significant contrast changes of the Co deposits during annealing to changes of the relative Co/C/O content (Figure 1a). In contrast, the as-deposited dark-brown Cu–C and Au–C deposits turned light-brown with temperature, suggesting that less significant changes occurred in the Cu–C(–O–F) and Au–C(–O) deposits during the post-growth treatment (Figure 1b,c).

**Figure 2:**
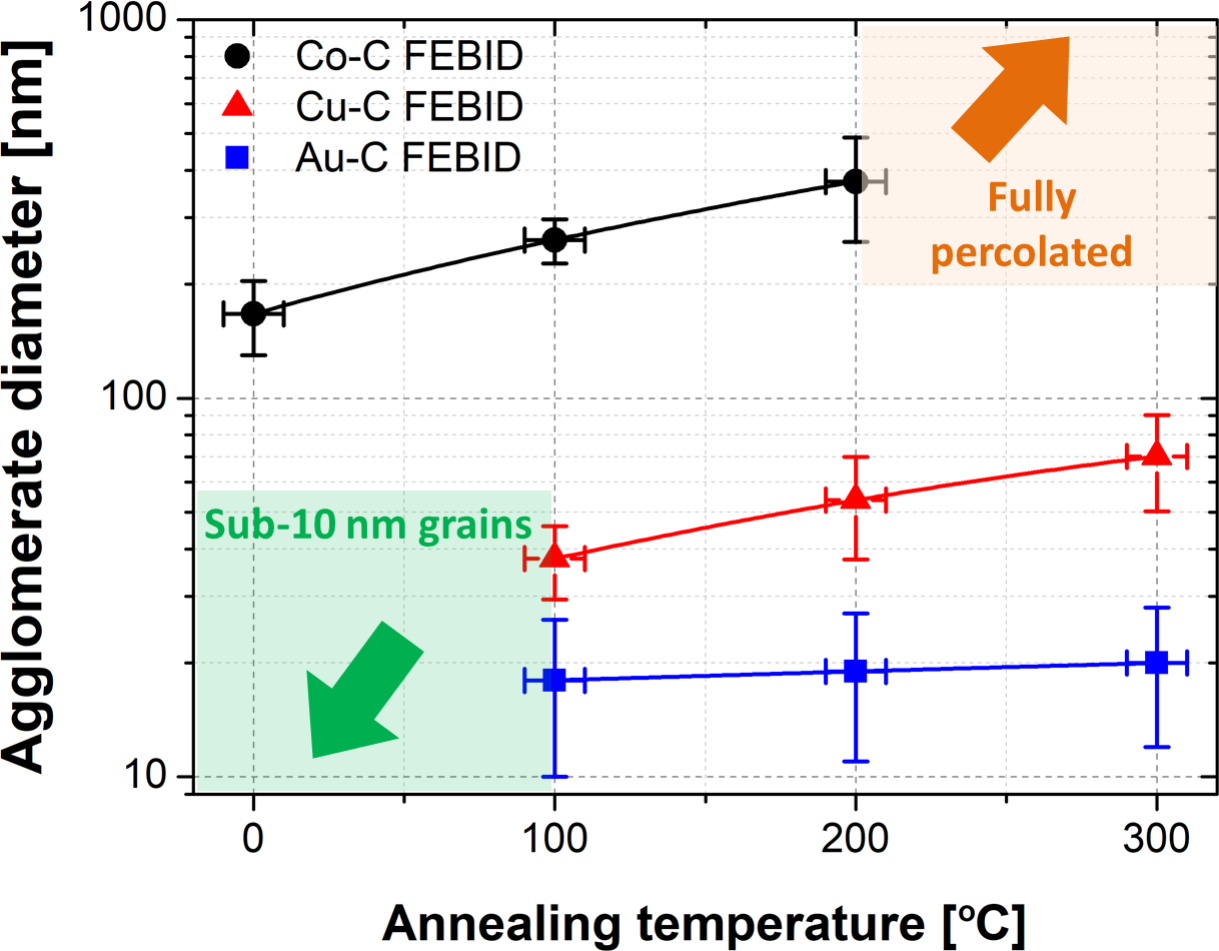
SEM-based average diameters of Co, Cu and Au FEBID agglomerates as function of the annealing temperature. At room temperature, Cu and Au agglomerates were visible in SEM but the contrast was too low to quantify the size. Instead, scanning transmission electron microscopy results from as-deposited materials are indicated. On the other hand, the Co grains fully percolate above 200 °C.

In order to obtain a more precise description of the induced changes, high-resolution imaging was performed using a Hitachi S-4800 SEM. In particular, the size of the metal agglomerates of all deposits was estimated from high-magnification SEM images (see Figure 1), using ImageJ software applying a fast Fourier transform (FFT) bandpass filter to enhance grain boundaries and a contrast-threshold algorithm to calculate the agglomerate diameter. It is worth mentioning that by SEM at high magnifications the whole metal agglomerates are detected rather than the individual grains forming the agglomerates. Individual grain (nanocrystal) sizes that can be determined by transmission electron microscopy are usually reported to be within a few nanometres for the FEBID materials under consideration, as well as to be pure metal [5,54–55]. Thus, the SEM measurements of the agglomerates rather give information on grain mobility and grain coalescence than on individual grain growth inside the agglomerates. Concerning the Co–C deposits, the granularity of the films and the border sharpness increase due to grain agglomeration. The extracted average size of the Co agglomerates more than doubles when the as-deposited FEBID material is annealed at 200 °C (from 170 ± 40 nm to 400 ± 100 nm, Figure 2). Increasing further the annealing temperature yields a conformal percolated metallic film (Figure 1a). On the other hand (see Figure 1b), for Cu–C deposits the annealing at 1000 °C leads to the formation of SEM-visible Cu agglomerations. As the temperature increases up to 300 °C, the agglomerates continue to grow reaching an average size twice as large as at 100 °C (33 ± 8 nm vs 70 ± 20 nm) while being dispersed inside and on top of the carbonaceous matrix. The Au–C deposits behave similarly except that the agglomerate size remains constant at 18 ± 8 nm above 100 °C. The input of thermal energy only leads to the appearance of a larger number of pure Au agglomerates inside and on top of the carbonaceous matrix.

### Chemical characterisation

#### EDX analysis

The atomic percentages of the constituent elements of the Co–C–O, Cu–C–F–O and Au–C–O FEBID materials were determined using energy-dispersive X-ray spectroscopy (EDX), performed using a Hitachi S-4800 SEM equipped with a silicon EDAX drift detector (SDD), acceleration voltage of 3 keV and take-off angle of ca. 32° over 100 s. The used emission current of 10 μA at 3 kV acceleration voltage yields a sample current of ca. 150 nA onto the substrate, extracted by a Faraday cup in the sample holder. Thus, the K-values of each atom were extracted from both the FEBID deposit and the substrate. The background signal from the detector, as well as the residual carbon signal arising from contamination deposition occurring during the EDX scan, were subtracted from the EDX spectra using a reference spectrum extracted far from the deposition area (see Supporting Information File 1). Finally, the SAMx STRATAGem software for thin film analysis was used to calculate the atomic composition of the FEBID deposits. In this way the EDX signal contribution from both the Si substrate and the 200 nm thick SiO_2_ layer underneath the deposits was correctly taken into account, ensuring an exact determination of the composition of the thin film FEBID materials within the errors of standardless quantification.

The variations of the elemental compositions differ between the three different FEB deposits investigated (Table 1). In particular, the Co–C FEBID material exhibits a strong variation in composition during thermal annealing. Improvements in Co content from 67 atom % for as-deposited films to 78 atom % at 100 °C and 84 atom % at 200 and 300 °C annealing temperature occurred predominantly due to oxygen release from the deposited material [5]. On the other hand, the Cu–C and Au–C FEBID materials did not exhibit composition variations during the annealing process (within the experimental error of 2 atom %). For Cu–C deposits, we obtain an approximately constant composition of Cu_0.06_C_0.42_O_0.52_. Earlier investigations performed at room-temperature-deposited and 200 °C annealed 150 nm thick Cu–C FEB square deposits (25 keV, 0.4 nA) from the same precursor also observed an almost constant copper content, yet slightly higher (10 ± 2 atom %) [27]. Also, these experiments found an oxygen decrease, from 25 to 13 atom %, concurrently with a carbon content increase, from 64 to 75 atom % with temperature. This behaviour was not reproduced in the present experiments. Furthermore, fluorine was not present in the current deposits, or its signal was below the EDX detection limit. In comparison, a fluorine content of 1 atom % at room temperature, which vanishes after annealing at 200 °C, was measured in the previous study [27].

**Table 1:** Summary of the atomic composition of the deposits measured by EDX within ± 2 atom % error.

	Co/C/O	Cu/C/O^a^	Au/C/O

as deposited	67:14:19	4:45:51	3:38:59
100 °C annealed	78:14:8	5:42:53	3:39:58
200 °C annealed	84:14:2	6:44:50	5:34:61
300 °C annealed	85:14:1	6:43:51	4:36:60

^a^The fluorine content of the Cu(hfac)_2_ FEBID deposit was found to be approximately zero or below the detection limit of EDX.

Similarly, the atomic compositions of the Au-containing FEBID material remained approximately constant at Au_0.05_C_0.36_O_0.59_ during annealing. This corroborates the findings of recent works that report the requirement of an oxidative atmosphere for a successful purification of noble metals such as Au and Pt [8,28,32,34]. Nevertheless, the gold content is low compared to previous experiments with the same precursor [55], which gave about 22 atom % Au. The main difference to the present experiments is that here we studied 50 nm thick thin films while the previous material was much thicker and received a higher dose during post-growth irradiation of the already deposited underlying material. The same mechanism could also be responsible for the lower copper content found in the present study.

#### Raman characterisation

Raman spectra were examined in the carbon range in order to elucidate the composition of the carbonaceous matrix during post-growth annealing. Raman spectroscopy was performed using an upright confocal Raman spectrometer (NT-MDT NTEGRA) featuring a laser source with a wavelength of 532 nm and a 250× objective with a numerical aperture of 0.90. Spectra were recorded at a spectral resolution of 2.7 cm^−1^, and the exposure time was 30 s for all samples.

**Figure 3:**
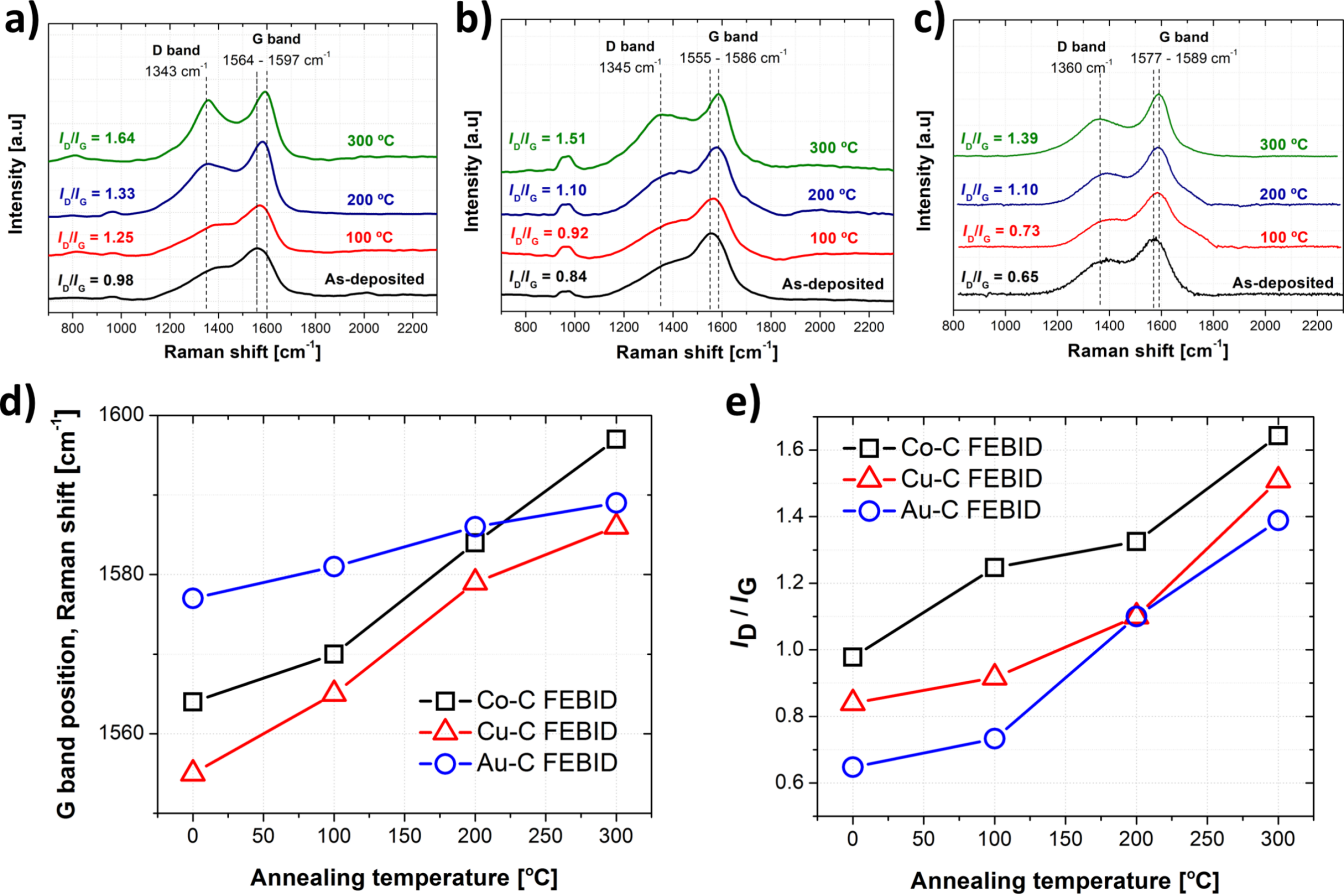
Top: Raman spectra in the carbon range as a function of the post-growth annealing temperature of (a) Co–C, (b) Cu–C and (c) Au–C deposits, showing the peaks of the disordered carbon band, D (1350 cm^−1^), as well as the graphitic band, G (1580 cm^−1^). Partial thermally-induced conversion of an amorphous carbon matrix into graphite nanocrystals in the FEBID material is indicated through (d) the shift of the G band peak position as a function of annealing temperature, as well as (e) of the integrated intensity ratio between D and G peaks, *I*_D_/*I*_G_.

Figure 3a–c present specific trends with respect to the carbon G peak shift and the integrated intensity ratio between the D and G peaks (*I*_D_/*I*_G_) (Figure 3d,e). The thermal conversion of amorphous carbon into graphite during FEBID using hydrocarbon compounds without a central metal atom has been previously reported by both Fedorov et al. [56] and Kulkarni and co-workers [57]. The rising D band peak accounts for the increase of boundaries, which turns the amorphous carbon into polycrystalline graphite. According to Tuinstra et al. [58], Robertson [59] and Ferrari and co-workers [60], both the G band shift and the integrated intensity ratio allow for the determination of the in-plane correlation length, or of the grain size of graphitic crystallites in a disordered carbon matrix.

The integrated intensities of individual D and G carbon bands were evaluated using a Lorentzian peak fitting in order to deconvolute them. Thus, the values of the *I*_D_/*I*_G_ ratio could be extracted as approximately 0.98, 0.84 and 0.65 for as-deposited Co–C, Cu–C and Au–C FEBID material, respectively, before monotonically increasing up to 1.64, 1.51 and 1.39, respectively, after annealing at 300 °C. According to the Tuinstra–Koenig relationship [58–59,61], values of around 0.5 represent mainly disordered (amorphous) carbon. Therefore, it mainly represents the carbon structure obtained for our as-deposited Au–C material, while some graphite clustering is already present in as-deposited Co–C and Cu–C. In addition, nanocrystalline graphite clustering is observed for the three FEBID materials after annealing at 300 °C. The calculated average nanocrystalline graphite cluster size remains around 13 nm for all deposits: 12 nm, 13 nm and 14 nm for Co–C, Cu–C and Au–C deposits, respectively.

Moreover, differences between the FEBID materials were observed with respect to the graphitisation process. Catalytic graphitisation using transition metals has been reported in literature [62], showing a superior graphitisation efficiency for Co as catalyst atom when compared to Cu. The slightly lower intensity ratios for Cu–C (average of around 8%), compared to Co–C, supports this claim. Furthermore, regarding the graphitisation in presence of Au, the lower integrated intensity ratios, as well as the lower G band peak shift (Figure 3c–e) indicate a minor influence of this type of metal on the graphitisation process (compared to Co and Cu) during annealing. It is worth mentioning that plasmon-assisted heating of the interface between amorphous carbon deposits and the substrate through low-power light irradiation enables the conversion of the amorphous phase into nanocrystalline graphite. Moreover, the degree of graphitisation strongly depends on the substrate material [63].

### Electrical transport characterisation

**Figure 4:**
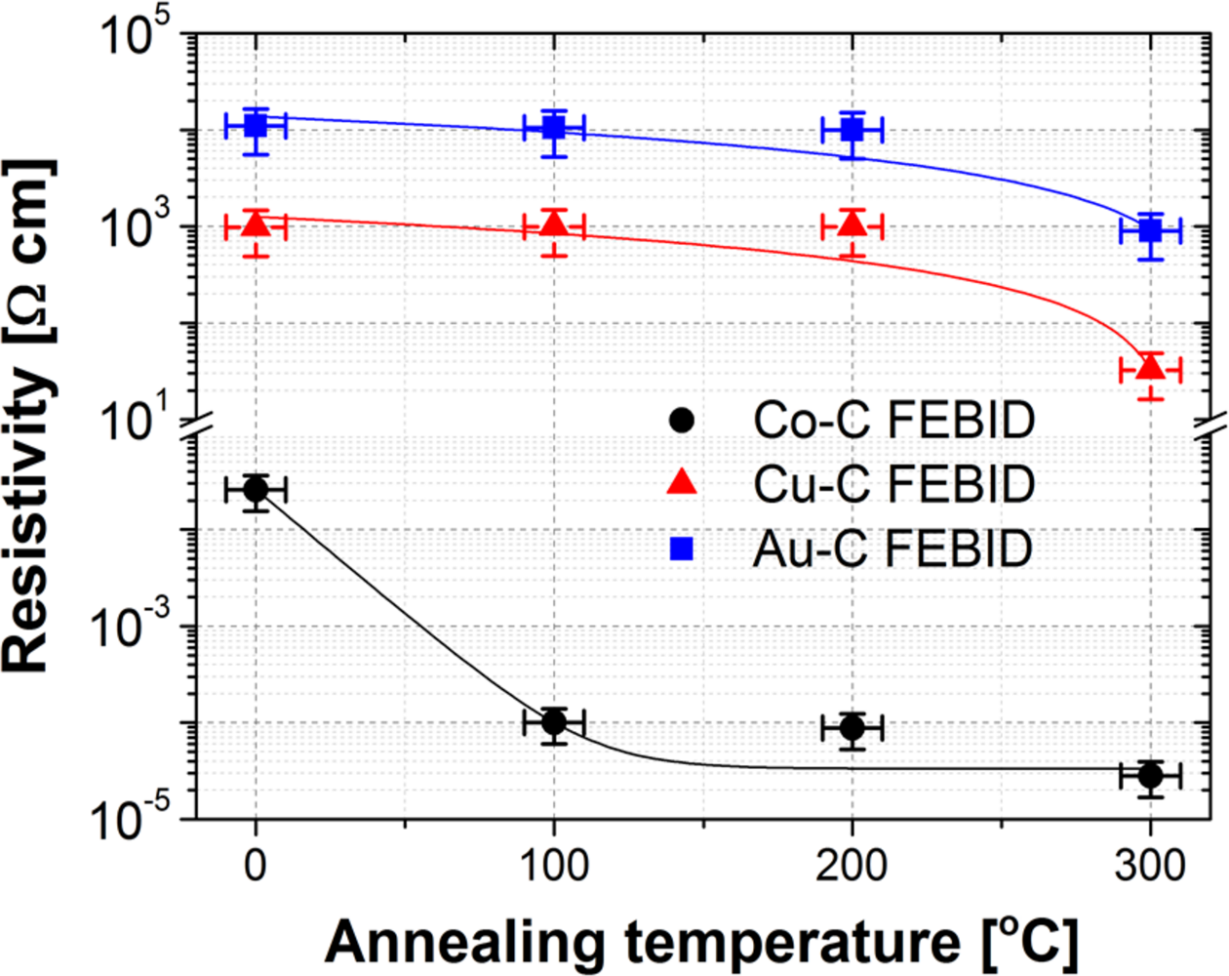
Electrical resistivities of Co–C, Cu–C and Au–C FEBID materials as a function of the annealing temperature, showing a monotonically decrease with temperature increase for all the deposits. Percolation of Co grains and release of oxygen are the main mechanisms for the reduction of resistivity of the Co samples. On the other hand, the graphitisation of carbon at temperatures higher than 200 °C is suggested as the main resistivity reduction mechanism in highly resistive deposits with low Cu and Au content.

The electrical resistances of the FEBID deposits were measured at room temperature using a conventional four-probe setup with a Keithley 2400 source-meter. Thus, the electrical resistivities could be calculated from the “*I* × *V*” curves (see Supporting Information File 1 for more details) with the deposit dimensions measured by AFM. We observed a monotonically decrease of the resistivities with annealing temperature (Figure 4). However, while the resistivity reduction of Co–C deposits is more pronounced within the first 100 °C of annealing (from 26 mΩ·cm to 0.1 mΩ·cm), the resistivities of Cu–C and Au–C remain approximately constant at 980 Ω·cm and 11000 Ω·cm, respectively, within 200 °C annealing. Increasing the annealing temperature up to 300 °C, the Co–C resistivity reduces only by a factor of three. In contrast, the Cu–C and Au–C resistivities reduce by factors of 30 (down to 32 Ω·cm) and 12 (down to 900 Ω·cm), respectively.

The large resistivity reduction observed in the Co–C deposits was already discussed in [5]. Due to the low metal content in both as-deposited Cu–C and Au–C FEBID materials, graphitisation plays an important role in the improvement of the conduction, occurring mainly at annealing temperatures larger than 200 °C. It is worth mentioning that a resistivity value of ca. 10^5^ Ω·cm is typically found in as-grown carbon material by FEBID [56]. We found, instead, values that are one and two orders of magnitude lower for Au–C and Cu–C, respectively, which may be attributed to the deposition regime which is close to electron-limited regime. Furthermore, according to Fedorov and co-workers [56], a nanocrystalline graphite deposit exhibits a resistivity of ca. 10^−4^ Ω·cm, which is about five to six orders of magnitude lower than the values found in our annealed samples. It suggests that only a partial thermally induced graphitisation was obtained for Cu–C and Au–C deposits under our experimental conditions.

Finally, the differences between Cu–C and Au–C are attributed to a higher graphite content in Cu–C deposits as observed in our Raman analysis. In addition, we attribute the resistivity difference of one order of magnitude between Cu–C and Au–C (Figure 4) to the larger oxygen content (ca. 6 atom %) in the latter material. Table 2 summarizes some of the deposits characteristics for a general overview and comparison between the precursors studied in this work.

**Table 2:** Property summary of the as-grown and 300 °C annealed FEBID materials.

	Co–C	Cu–C	Au–C

metal content, atom % (as grown / 300 °C)	67 / 85 ± 2	4 / 6 ± 2	3 / 4 ± 2
electrons/precursor molecule ratio	0.6 ± 0.1	8.9 ± 0.2	7.2 ± 0.1
graphite crystal size, nm (as grown / 300 °C)	<1 / 13	<1 / 12	<1 / 14
metal agglomerate size, nm (as grown / 300 °C)	170 ± 40 / 400 ± 100	<1 / 70 ± 20	<1 / 18 ± 8
resistivity, Ω·cm (as grown / 300 °C)	26 × 10^−3^ / 26 × 10^−6^	980 / 32	11 × 10^3^ / 900
thickness shrinkage, %	7–10%	<5%	<5%

### H_2_-assisted post-growth purification of Au-C deposits

**Figure 5:**
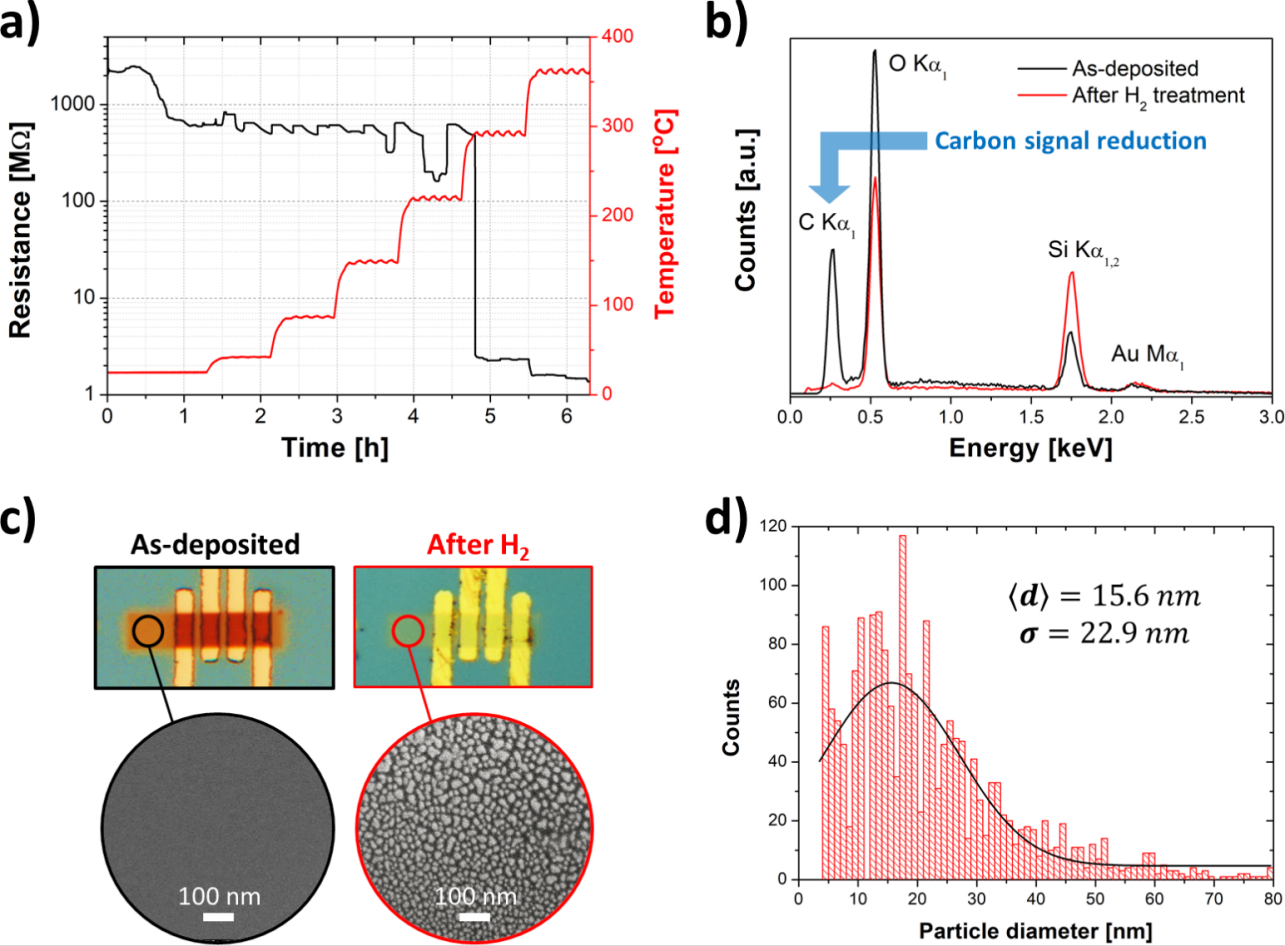
(a) Time-evolution of the electrical resistance during annealing in a 200 ppm H_2_ atmosphere, revealing a resistance reduction of about three orders of magnitude at 360 °C. (b) EDX spectra of as-deposited and annealed samples, presenting a drastic carbon signal reduction down to values close to the carbon background signal from the chamber, thus highlighting the efficiency of the H_2_-based purification method. Silicon and oxygen signals originate from the SiO_2_/Si substrate. (c) Optical (top) and scanning electron (bottom) microscopies of both as-deposited and annealed at 360 °C Au–C FEBID material. This annealing procedure efficiently removes the carbon matrix allowing for the nucleation of pure Au grains with (d) around 16 nm average size and 23 nm dispersion.

A H_2_-assisted purification annealing procedure was carried out for as-deposited Au-C in a 200 ppm H_2_ environment heated from room temperature to 360 °C over a period of 6.5 h. During the annealing procedure, the temperature was increased by about 6 °C·min^−1^ and then kept constant for 40 min before a new temperature increment (Figure 5a). The electrical resistance, which was monitored with a two-wire setup, monotonically decreased three orders of magnitude during this annealing procedure. The initial resistance of 2 GΩ decreased down to around 500 MΩ below 300 °C, where it abruptly decreases to 2 MΩ. Then, as the temperature continues to increase, the resistance decreases, finally assuming the lowest value of 1.5 MΩ at a temperature of 360 °C after 5.5 h. Afterwards, the atomic composition was measured by EDX (Figure 5b). It shows a drastic reduction of the carbon content to values close to the background signal from the chamber. It can be attributed to the thermally induced formation of hydrocarbons driven by a Fischer–Tropsch-like reaction [64]. The hydrocarbons are volatile and escape from the FEBID material above 300 °C.

Figure 5c presents optical and scanning electron microscopies of the as-written and H_2_-assisted purified deposits. A colour change from brown (carbon-rich) to light-yellow (purified) was observed by optical microscopy. In addition, big structural changes were observed with respect to the grain size upon the thermal treatment. Sub-10 nm grains dispersed in a carbonaceous matrix (as-deposited material) turn into larger nucleated pure Au crystals with an average size of 15.6 nm (Figure 5d), after annealing under H_2_ atmosphere.

We believe that further electrical conductivity improvements may be achieved with thicker Au–C deposits, as the larger metallic mass will contribute to form a fully percolated metallic film with much lower resistance.

## Conclusion

We have used a vacuum-based post-growth annealing protocol for tuning the crystalline properties of FEBID materials produced under a low electron-beam density from non-noble metal, Co_2_(CO)_8_ and Cu(hfac)_2_, as well as noble metal, Me_2_Au(acac), precursors. The as-deposited Cu–C and Au–C FEBID materials formed SEM-visible metal agglomerates of Cu and Au at around 100 °C. After annealing at 300 °C, the average metal agglomerate size was 70 ± 20 nm for Cu and 18 ± 8 nm for Au. EDX analysis showed no dependence on temperature of the atomic composition of copper- and gold-containing FEBID materials (around 4–6 atom % metal), suggesting that during annealing no volatile compounds are formed. In contrast, the as-deposited Co–C FEBID material showed a high metal content of 67 atom %. During post-growth annealing mainly oxygen is released while the carbon content remains approximately constant. The cobalt agglomerates coalesce to form a compact metallic film. Regardless of the chemical difference of the three precursors and the deposit composition, graphitisation of the amorphous carbon matrix was observed by Raman analysis in all cases.

Electrical measurements show a reduction of the resistivity of Co–C by two orders of magnitude after annealing at 100 °C, which can be attributed to the coalescence of metallic agglomerates and the release of oxygen. On the other hand, the resistivities of Cu–C and Au–C remain approximately constant up to 200 °C annealing temperature, and decrease about one order of magnitude due to graphitisation of amorphous carbon at 300 °C. The larger resistivity reduction of the Cu–C deposits (a factor of 2.5 with respect to Au–C) is attributed to a better graphitisation mechanism.

Overall, the tuning protocols presented in this work proved to be exceptionally reproducible and are expected to be adequate for applications that require the definition of sub-10 nm structures. This is possible, as the nonthermal electron-induced deposition with low electron densities guarantees lateral resolution fidelity, while the annealing process allows for tuning the material properties and results in a graphitic matrix that protects the metal against atmospheric oxidation. The new H_2_-assisted purification procedure efficiently removed the carbon matrix of as-deposited Au–C FEBID materials at temperatures above 300 °C, allowing for a resistance reduction of three orders of magnitude. The resulting material comprises pure nucleated Au grains with around 16 nm average size.

## Supporting Information

File 1 Additional experimental data.
