# Supplementary material for: Comparative study of post-growth annealing of Cu(hfac)2, Co2(CO)8 and Me2Au(acac) metal precursors deposited by FEBID
Source: Beilstein J Nanotechnol. 2018 Jan 9;9:91–101. doi: 10.3762/bjnano.9.11 (PMC5789384; doi:10.3762/bjnano.9.11)
Supplement: File 1 — Additional experimental data. [file Beilstein_J_Nanotechnol-09-91-s001.pdf]

Supporting Information

for

Comparative study of post-growth annealing of $Cu(hfac)_2$, $Co_2(CO)_8$ and $Me_2Au(acac)$ metal precursors deposited by FEBID

Marcos V. Puydinger dos Santos*[1,2,3], Aleksandra Szkudlarek[4], Artur Rydosz[5], Carlos Guerra-Nuñez[3], Fanny Béron[1], Kleber R. Pirota[1], Stanislav Moshkalev[2], José Alexandre Diniz[2] and Ivo Utke*[3]

Address: [1]Institute of Physics Gleb Wataghin, University of Campinas, Rua Sérgio Buarque de Holanda 777 Cidade Universitária, 13083-859, Campinas-SP, Brazil, [2]Faculty of Electrical and Computing Engineering and Center for Semiconductor Components and Nanotechnologies, University of Campinas, Rua Pandiá Calógeras 90, Cidade Universitária, 13083-870, Campinas-SP, Brazil. [3]Laboratory for Mechanics of Materials and Nanostructures, Swiss Federal Laboratories for Materials Science and Technology (EMPA), Feuerwerkerstrasse 39, 3602 Thun, Switzerland, [4]AGH University of Science and Technology, Academic Centre for Materials and Nanotechnology, al. A. Mickiewicza 30, 30-059 Krakow, Poland and [5]AGH University of Science and Technology, Faculty of Computer Science, Electronics and Telecommunications, Av. Mickiewicza 30, 30-059 Krakow, Poland

Email: Marcos Vinicius Puydinger dos Santos - puyding@ifi.unicamp.br;
Ivo Utke – Ivo.Utke@empa.ch

* Corresponding author

Additional experimental data

Suppl. 1 – EDX analysis

Energy-dispersive X-ray spectroscopy (EDX) was carried out using a Hitachi S-4800 SEM equipped with a silicon EDAX drift detector (SDD), acceleration voltage of 3 keV electrons and take-off angle of ca. 32° during 100 s. The used emission current of 10 μA at a 3 kV acceleration voltage yields a sample current of ca. 150 nA onto the substrate, extracted by a Faraday cup in the sample holder. Thus, the K-values of each atom were extracted from both the FEBID deposit and the substrate. The background signal from the detector, as well as the residual carbon signal arising from contamination deposition occurring during the EDX scan, were subtracted from the EDX spectra using a reference spectrum extracted far from the deposition area (**Figure S1**). Finally, SAMx STRATAGem software for thin film analysis was used to calculate the atomic composition of the thin FEBID deposits from the corrected spectra (**Figure S2**). This way the EDX signal contribution from both the Si substrate and the 200 nm-thick SiO_2 layer underneath the deposits were correctly taken into account, ensuring a reliable estimation of the thin film FEBID material composition.

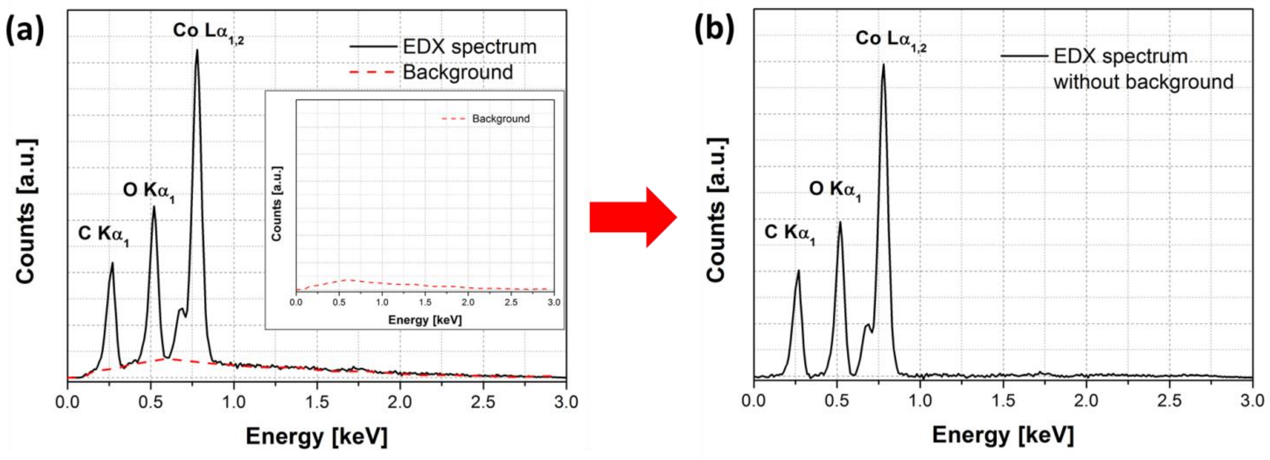

Figure S1. Example of EDX spectrum taken with 3 keV electrons (a) from 35 nm-thick Co FEBID and (b) after background removal prior to composition analysis. This illustrates the procedure utilized in this work for the residual carbon contamination, as well as detector background removal from all the deposits.

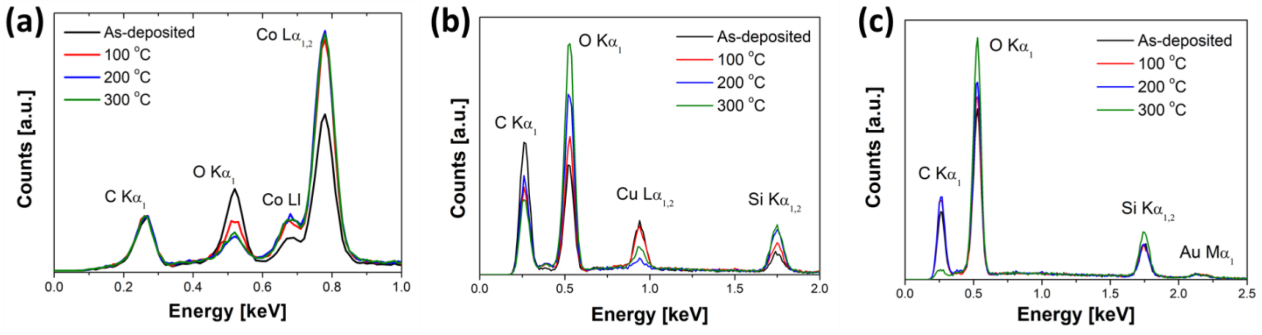

Figure S2. EDX spectra taken with 3 keV electrons from (a) 35 nm-thick Co-C FEBID and 50 nm-hick (b) Cu-C FEBID and (c) Au-C FEBID after background removal prior to composition analysis by STRATAGEM.

Suppl. 2 – I x V curves

The deposits electrical resistances were extracted at room temperature using a conventional four-probe setup with a Keithley 2400 source-meter. In order to extract the I x V curves, currents from hundreds of nA up to a few µA were driven while the voltages could be read (with a compliance set at 20 V). This method was chosen to keep the power dissipation in the deposits in the range of µW, as well as preserving them in case of resistance reduction due to Joule heating. Therefore, the power dissipation always decreases with a resistance reduction.

Figure S3 shows I x V of as-deposited and 100 °C, 200 °C and 300 °C annealed Co-C FEBID material. For all cases a linear behavior is present. In addition, **Figure S4** shows both I x V curves of the 300 °C annealed Cu-C and Au-C deposits, which correspond to a linear behavior as well. The resistance values are, respectively, 5 and 6 orders of magnitude larger than that for the 300 °C annealed Co-C FEBID material. Furthermore, the I x V curves for as-deposited and annealed at 100 °C and 200 °C Cu-C and Au-C deposits showed a non-linear (Schottky-like) behavior with extremely large resistance values (around 42 MΩ and 386 MΩ, respectively) which were then calculated driving a constant current of 100 nA while reading the average voltage over 100 samples.

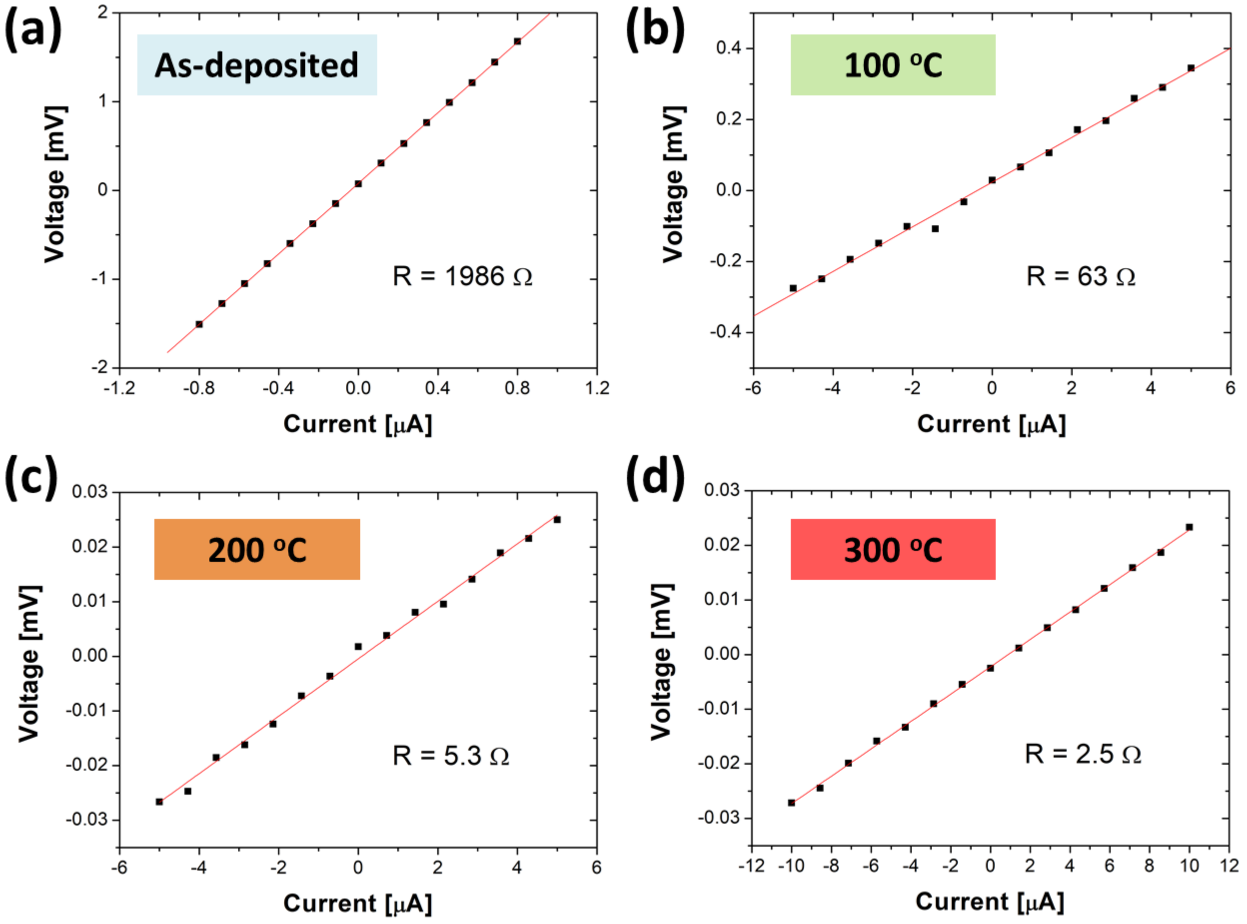

Figure S3. I x V curves of (a) as-deposited and annealed at (b) 100 °C, (c) 200 °C and (d) 300 °C Co-C FEBID material.

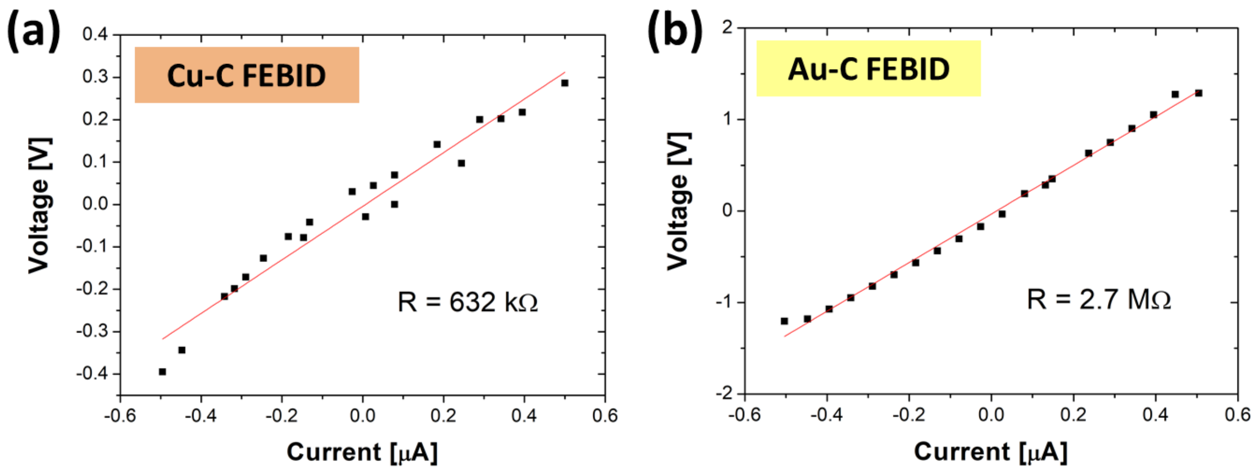

Figure S4. I x V curves of 300 °C annealed (a) Cu-C and (b) Au-C FEBID material.
